# Anti–NF-**κ**B peptide derived from nuclear acidic protein attenuates ovariectomy-induced osteoporosis in mice

**DOI:** 10.1172/jci.insight.171962

**Published:** 2023-11-22

**Authors:** Kenji Takami, Kazuki Okamoto, Yuki Etani, Makoto Hirao, Akira Miyama, Gensuke Okamura, Atsushi Goshima, Taihei Miura, Takuya Kurihara, Yuji Fukuda, Takashi Kanamoto, Ken Nakata, Seiji Okada, Kosuke Ebina

**Affiliations:** 1 Department of Orthopaedic Surgery, Osaka University Graduate School of Medicine, Suita, Osaka, Japan.; 2 Department of Orthopaedic Surgery, Nippon Life Hospital, Nishi-ku, Osaka, Japan.; 3 Department of Obstetrics and Gynecology, Osaka University Graduate School of Medicine, Suita, Osaka, Japan.; 4 Department of Orthopaedic Surgery, National Hospital Organization Osaka Minami Medical Center, Kawachinagano, Osaka, Japan.; 5 Department of Orthopaedic Surgery, National Hospital Organization Osaka Toneyama Medical Center, Toyonaka, Osaka, Japan.; 6 Department of Orthopaedic Surgery, Osaka Rosai Hospital, Kita-ku, Sakai, Japan.; 7 Department of Health and Sport Sciences, and; 8 Department of Musculoskeletal Regenerative Medicine, Osaka University Graduate School of Medicine, Suita, Osaka, Japan.

**Keywords:** Bone Biology, Mouse models, NF-kappaB, Osteoporosis

## Abstract

NF-κB is a transcription factor that is activated with aging. It plays a key role in the development of osteoporosis by promoting osteoclast differentiation and inhibiting osteoblast differentiation. In this study, we developed a small anti–NF-κB peptide called 6A-8R from a nuclear acidic protein (also known as macromolecular translocation inhibitor II, Zn^2+^-binding protein, or parathymosin) that inhibits transcriptional activity of NF-κB without altering its nuclear translocation and binding to DNA. Intraperitoneal injection of 6A-8R attenuated ovariectomy-induced osteoporosis in mice by inhibiting osteoclast differentiation, promoting osteoblast differentiation, and inhibiting sclerostin production by osteocytes in vivo with no apparent side effects. Conversely, in vitro, 6A-8R inhibited osteoclast differentiation by inhibiting NF-κB transcriptional activity, promoted osteoblast differentiation by promoting Smad1 phosphorylation, and inhibited sclerostin expression in osteocytes by inhibiting myocyte enhancer factors 2C and 2D. These findings suggest that 6A-8R has the potential to be an antiosteoporotic therapeutic agent with uncoupling properties.

## Graphical abstract

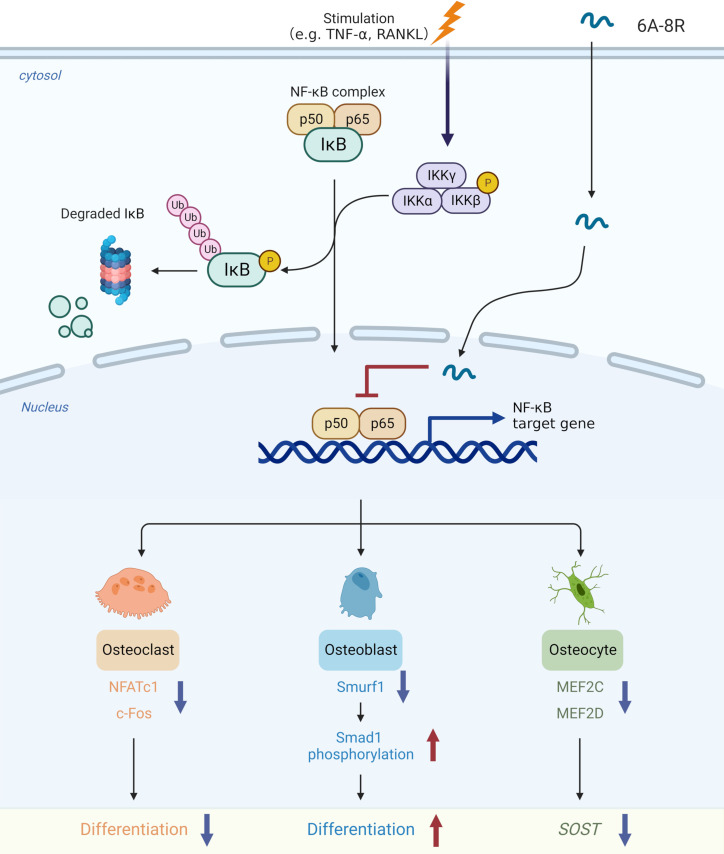

## Introduction

Bone homeostasis is primarily maintained by the cooperation of osteoblasts and osteoclasts — a process known as bone coupling (1, 2). Bisphosphonates and denosumab are commonly used antiosteoporotic drugs. They mainly inhibit bone resorption by osteoclasts, although they also inhibit bone formation by osteoblasts via by inhibiting coupling. Therefore, the prolonged use of these drugs may increase the risk of serious adverse events such as osteonecrosis of the jaw and atypical femoral fractures (3). Regarding bone anabolic agents, teriparatide increases bone formation as well as bone resorption, leading to cortical porosity and transient loss of bone mineral density of the hip (4). Romosozumab is the newest bone anabolic agent that shows uncoupling bone effects by promoting bone formation and inhibiting bone resorption through Wnt signaling activation. However, due to concerns regarding cardiovascular events, the United States Food and Drug Administration warned against using romosozumab to treat patients with a history of cardiovascular events within 1 year (5). Thus, there remains a strong need for safer bone-uncoupling agents with other mechanisms of action.

Receptor activator of NF-κB ligand (RANKL) is widely known to play a crucial role in osteoclastogenesis (6–11). The expression of its receptor, which is the receptor activator of NF-κB (RANK), is induced in osteoclast precursors by macrophage colony–stimulating factor (M-CSF) (12–14). RANKL binds to RANK and activates various signaling cascades, and it consequently activates the NF-κB cascade and nuclear factor of activated T cells 1 (NFATc1), which is the main transcription factor of osteoclast differentiation (15).

Furthermore, NF-κB plays an inhibitory role in the proliferation and differentiation of osteoblasts by inducing Smurf1 activation, which increases Smad degradation (16–19), and by promoting the degradation of runt-related transcription factor 2 (Runx2) and β-catenin (20–23). Thus, NF-κB inhibition is considered a potential uncoupling therapeutic target that promotes bone formation and inhibits bone resorption.

Although there are several previous studies on NF-κB cascade inhibitors, none of the NF-κB cascade inhibitors has yet been used in clinical settings (24, 25). This is because most of the previous studies reported inhibition of the NF-κB pathway upstream of the cascade, which may lead to side effects such as hepatocyte apoptosis, hepatocellular carcinoma, inflammation of the colon, and immune system abnormalities (24, 26–32).

In our previous study, we found that macromolecular translocation inhibitor II (MTI-II) (33) — also known as Zn^2+^-binding protein (34) or parathymosin (35) — inhibits the transcriptional activity of NF-κB by directly binding to NF-κB after stimulation by tumor necrosis factor-α (TNF-α) (36). Furthermore, we synthesized 40A-8R, a peptide composed of 40 amino acids of the effector site of MTI-II fused with 8 arginine residues in the C-terminus (36). Oligoarginines were added to amino acids because arginine-rich peptides can be efficiently internalized into cells, and they are widely used as carriers for the intracellular delivery of bioactive molecules (37–39).

Systemic TNF-α levels were found to be elevated in ovariectomized mice (40–48), and TNF-α activates osteoclasts via NF-κB and promotes sclerostin production in osteocytes (49–51). Moreover, it has been reported that the activation of NF-κB inhibits osteoblast differentiation. Therefore, we hypothesized that MTI-II and related molecules are good candidates for bone-uncoupling antiosteoporotic treatment for ovariectomy-induced (OVX-induced) osteoporosis. However, long peptides, such as 40A-8R, are expensive; thus, their clinical application was considered unfeasible. Hence, we decided to create a small peptide molecule, the molecular size of which may be advantageous in terms of facilitating synthesis and reducing immunogenicity of the peptide, although 40A-8R showed no immune epitope based on a search of the immune epitope database (https://www.iedb.org/).

In the present study, we synthesized a peptide (6A-8R) similar to 40A-8R in terms of NF-κB inhibition but with a smaller molecular weight than 40A-8R. This study aimed to investigate the safety, efficacy, and molecular mechanisms of 6A-8R in OVX-induced osteoporosis in mice.

## Results

### Construction of anti–NF-κB peptide.

**Figure 1:**
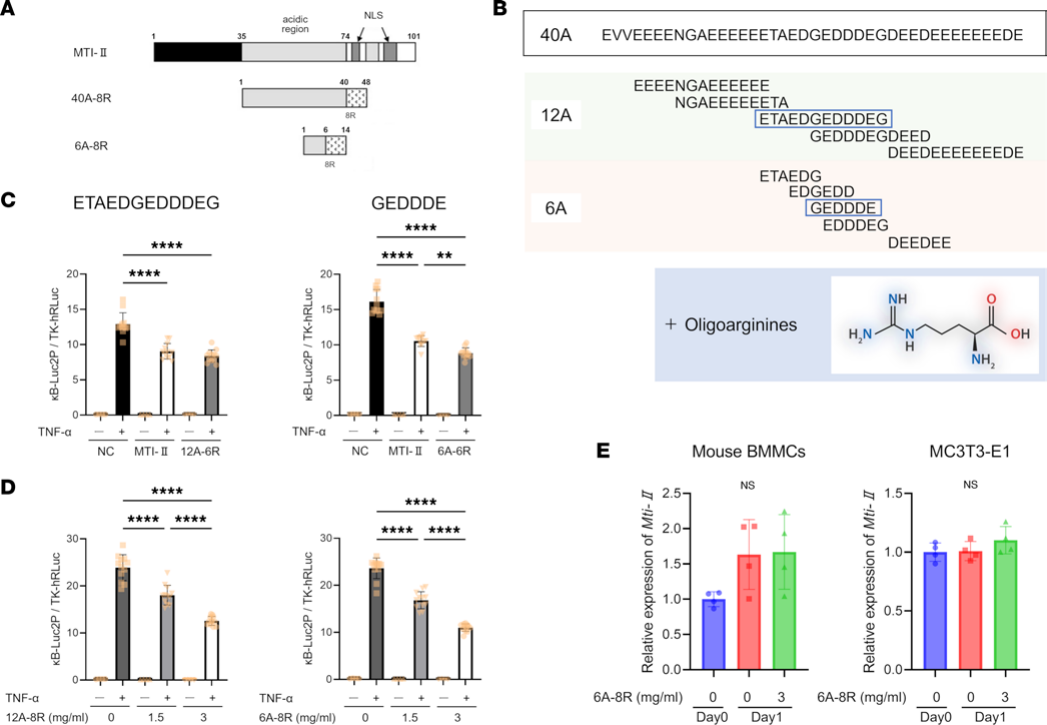
Preparation of MTI-II–based anti–NF-κB drugs. (**A**) Schematic representations of MTI-II, 40A-8R, and 6A-8R. A, amino acids; R, oligoarginine residues; NLS, nuclear localization signal. (**B**) Amino acid sequence of 40A and the candidate sequences of 12A and 6A in the active site. The 2 effector sequences are enclosed in a box. Monotonous runs of 6 or 8 arginine residues (6R or 8R) were added to the C-terminal region of each peptide. (**C**) NF-κB–induced luciferase activity was measured in HeLa cells transfected with MTI-II, 12A-6R, and 6A-6R expression vectors along with 2 luciferase reporter genes (κB-Luc2P and TK-hRLuc). Luciferase activity was measured after stimulation with TNF-α (1 ng/mL). Data are expressed as a ratio of κB-Luc2P activity to TK-hRLuc activity (internal control) and are presented as mean ± SD (*n* = 4 without TNF-α, *n* = 12 with TNF-α). NC, negative (empty vector) control. (**D**) NF-κB–induced luciferase activity was measured in HeLa cells transfected with luciferase reporter genes (κB-Luc2P and TK-hRLuc). After 10 hours of transfection, the cells were cultured with each concentration of 12A-8R and 6A-8R for 24 hours; subsequently, TNF-α (1 ng/mL) was added. Luciferase activity was measured 4.5 hours after stimulation with TNF-α. Data are expressed as a ratio of κB-Luc2P activity to TK-hRLuc activity (internal control) and are presented as mean ± SD (*n* = 4 without TNF-α, *n* = 12 with TNF-α). (**E**) Quantitative real-time PCR of mouse bone marrow mononuclear cells (BMMCs) and MC3T3-E1 cells. The relative gene expression of *Mti-II* with or without differentiating stimulations and 6A-8R (3 mg/mL) is plotted on the *y* axis. Data were statistically analyzed using 1-way ANOVA and Tukey-Kramer test. **P < 0.01; ****P < 0.0001. NS, not significant.

Our previous study reported that 40A-8R — developed from the effector site of MTI-II (Figure 1A) — shows antiinflammatory effects in various animal models of inflammatory diseases (36).

We further selected 10 candidates of the NF-κB inhibitor (Figure 1B) and made the expression vectors of these candidates with a 6-arginine residue (6R) sequence in the C-terminus. Transfections of these candidates into HeLa cells revealed that 2 out of the 10 candidates have NF-κB inhibitory activity (the effector sequences are enclosed in a box in Figure 1B). Figure 1C shows the effects of using the 2 candidates and MTI-II in the inhibition of NF-κB transcriptional activity. Supplemental Figure 1 (supplemental material available online with this article; https://doi.org/10.1172/jci.insight.171962DS1) shows the effects of using the other 8 sequences that were less effective in suppressing NF-κB. In contrast, the addition of 12A-6R and 6A-6R peptides had no effect on NF-κB transcriptional activity (data not shown). Thus, we hypothesized that 6 arginine residues may be insufficient to internalize these peptides into cells, and we explored peptides with 8 arginine residues (8R) to improve its cell-penetrating effect. Figure 1D shows that both 12A-8R and 6A-8R peptides (1.5 and 3.0 mg/mL) inhibit NF-κB transcription. These findings suggest that 8R (not 6R) is required to internalize the peptide into cells to show its efficacy. Furthermore, the efficacy was confirmed by Western blotting to detect cyclooxygenase 2 (COX2) protein expression in HeLa cells stimulated with TNF-α (1.0 ng/mL) (Supplemental Figure 2).

### Expression of MTI-II mRNA in osteoblasts and osteoclasts.

The quantitative real-time polymerase chain reaction (PCR) was used to determine the extent to which MTI-II mRNA was expressed in osteoclasts (mouse bone marrow mononuclear cells [BMMCs]) and osteoblasts (MC3T3-E1 cells) before and after inducing differentiation with or without 6A-8R (3.0 mg/mL; Figure 1E).

In mouse BMMCs, MTI-II gene expression tended to increase with the induction of differentiation. Furthermore, the gene was well expressed in MC3T3-E1 cells, regardless of differentiation induction. Moreover, the presence of 6A-8R (3.0 mg/mL) did not affect the expression levels of MTI-II in each cell type during differentiation induction (Figure 1E).

### Effects of 6A-8R on ovariectomized mice.

**Figure 2:**
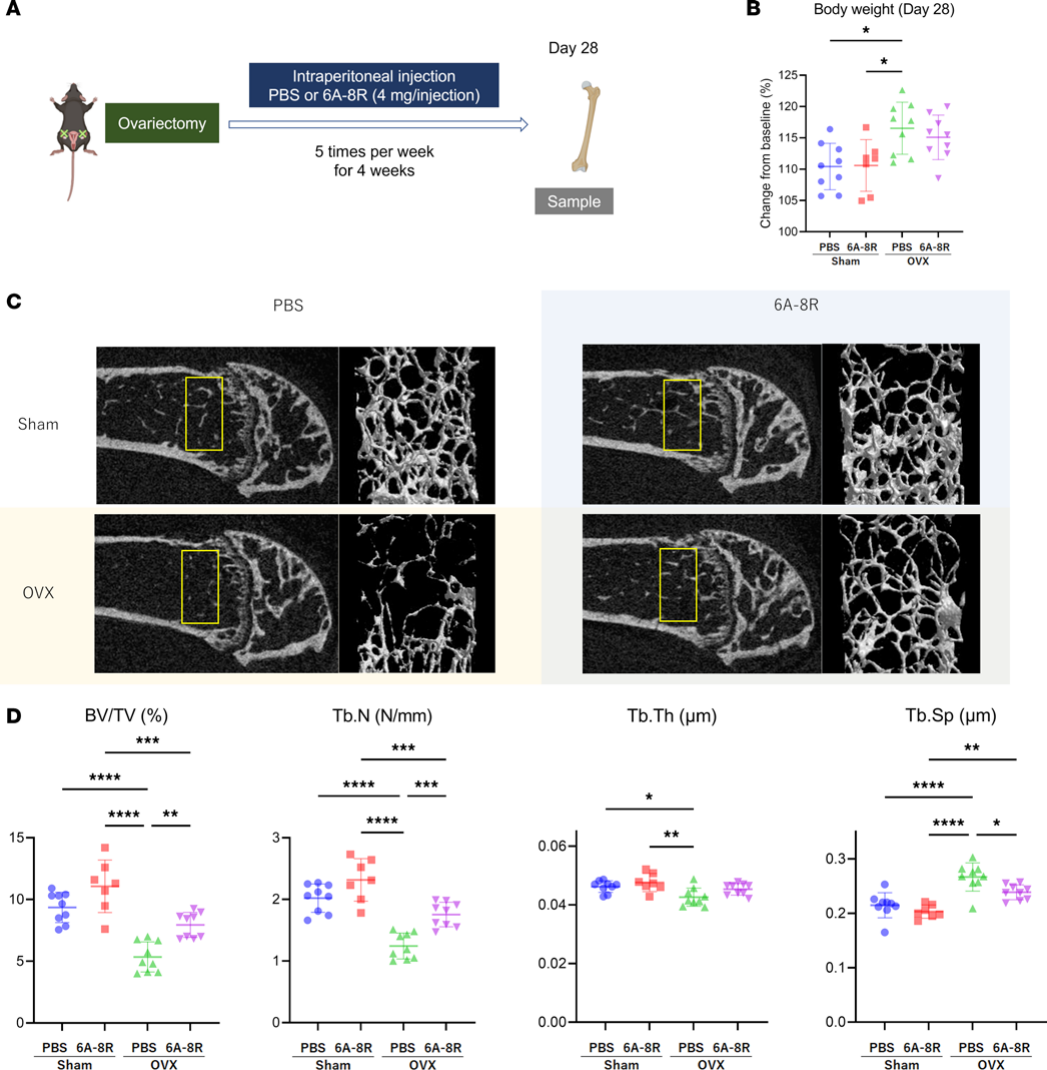
Effects of 6A-8R on ovariectomized (OVX) mice. (**A**) Schematic protocol of the animal experiment. Four milligrams of 6A-8R was intraperitoneally administered 5 days per week for 4 weeks, and samples (femurs) were collected. (**B**) Percentage changes in the body weight of the mice from baseline in each group. (**C**) Micro-CT images of the distal part of the femur on day 28 after OVX with or without 6A-8R administration. (**D**) Cancellous bone volume (BV)/tissue volume (TV), trabecular number (Tb.N), trabecular thickness (Tb.Th), and trabecular separation (Tb.Sp). Data are expressed as mean ± SD (*n* = 7 or 9) and were statistically analyzed using 1-way ANOVA and the Tukey-Kramer test. *P < 0.05; **P < 0.01; ***P < 0.001; ****P < 0.0001.

To evaluate the effects of administering 6A-8R to ovariectomized mice (Figure 2A), we first investigated the effect of 6A-8R on mouse body weight and found no significant differences between before and after 6A-8R administration (Figure 2B). Furthermore, following 6A-8R administration, there was no obvious damage to the liver or kidneys (Supplemental Figure 3).

For 4 weeks, 4 mg of 6A-8R was administered intraperitoneally for 5 days per week, and samples (femurs) were collected. The administration dose of 6A-8R was determined based on a previously described study (see Methods) (36).

Figure 2C shows representative results of micro-computed tomography (micro-CT) of cancellous bone in the distal part of the femur on day 28. The OVX groups had significantly lower bone volume (BV)/tissue volume (TV), trabecular number (Tb.N), and trabecular thickness (Tb.Th) of cancellous bone, and had significantly higher trabecular separation (Tb.Sp). The administration of 6A-8R significantly improved these parameters and tended to improve Tb.Th (Figure 2D). The findings regarding BV exhibited similarity to BV/TV, while no significant differences were observed among the groups in terms of bone marrow density (BMD) and cortical bone parameters (total area [Tt.Ar], cortical area [Ct.Ar], and Ct.Ar/Tt.Ar) (Supplemental Figure 4).

**Figure 3:**
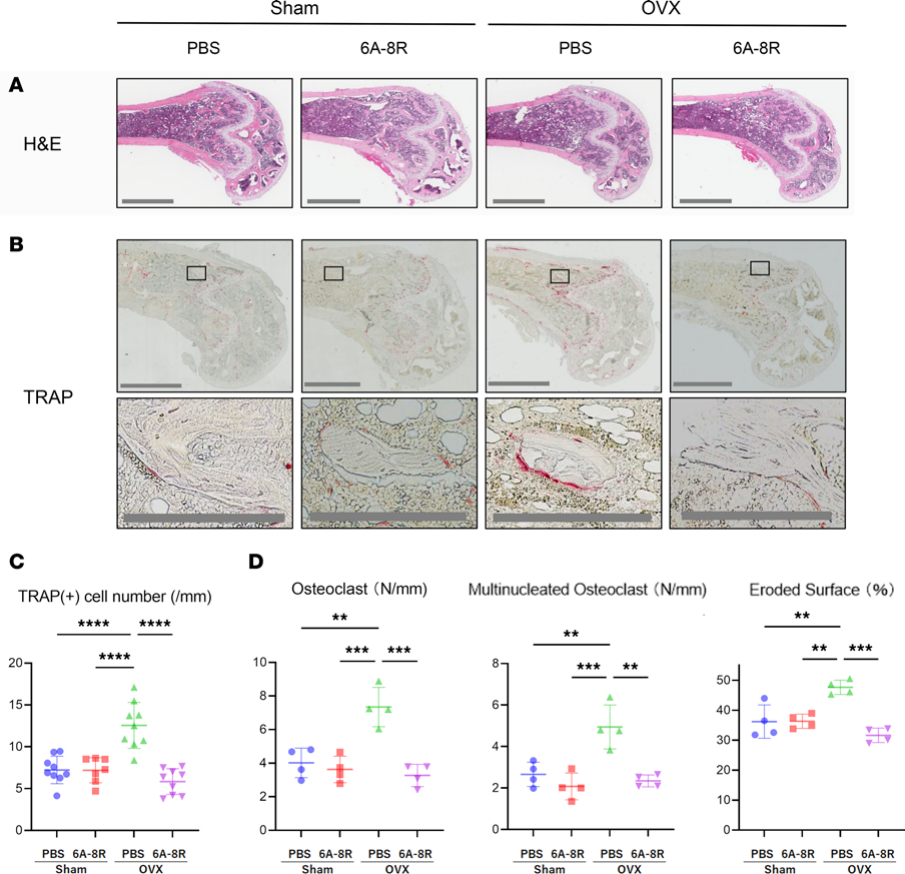
Histological and histomorphometric analysis of the distal part of the femur for osteoclasts in Sham-operated and OVX mice with or without intraperitoneal injection of 6A-8R (4 mg) 5 days per week for 4 weeks. (**A**) Histological findings of the distal part of the femur stained with hematoxylin and eosin. Scale bar: 1 mm. (**B**) TRAP staining. Scale bars: 1 mm (top and middle rows) and 200 μm (bottom row). (**C**) Plot of the number of TRAP-positive cells per unit trabecular surface. Data are expressed as mean ± SD (*n* = 7 or 9). (**D**) Histomorphometric findings of the distal part of the femur. Plots of the number of osteoclasts (Oc.N) (N/mm), number of multinucleated osteoclasts (M.Oc.N) (N/mm), and eroded surface (ES)/bone surface (BS) (%). Data are expressed as mean ± SD (*n* = 4) and were statistically analyzed using 1-way ANOVA and the Tukey-Kramer test. **P < 0.01; ***P < 0.001; ****P < 0.0001.

**Figure 4:**
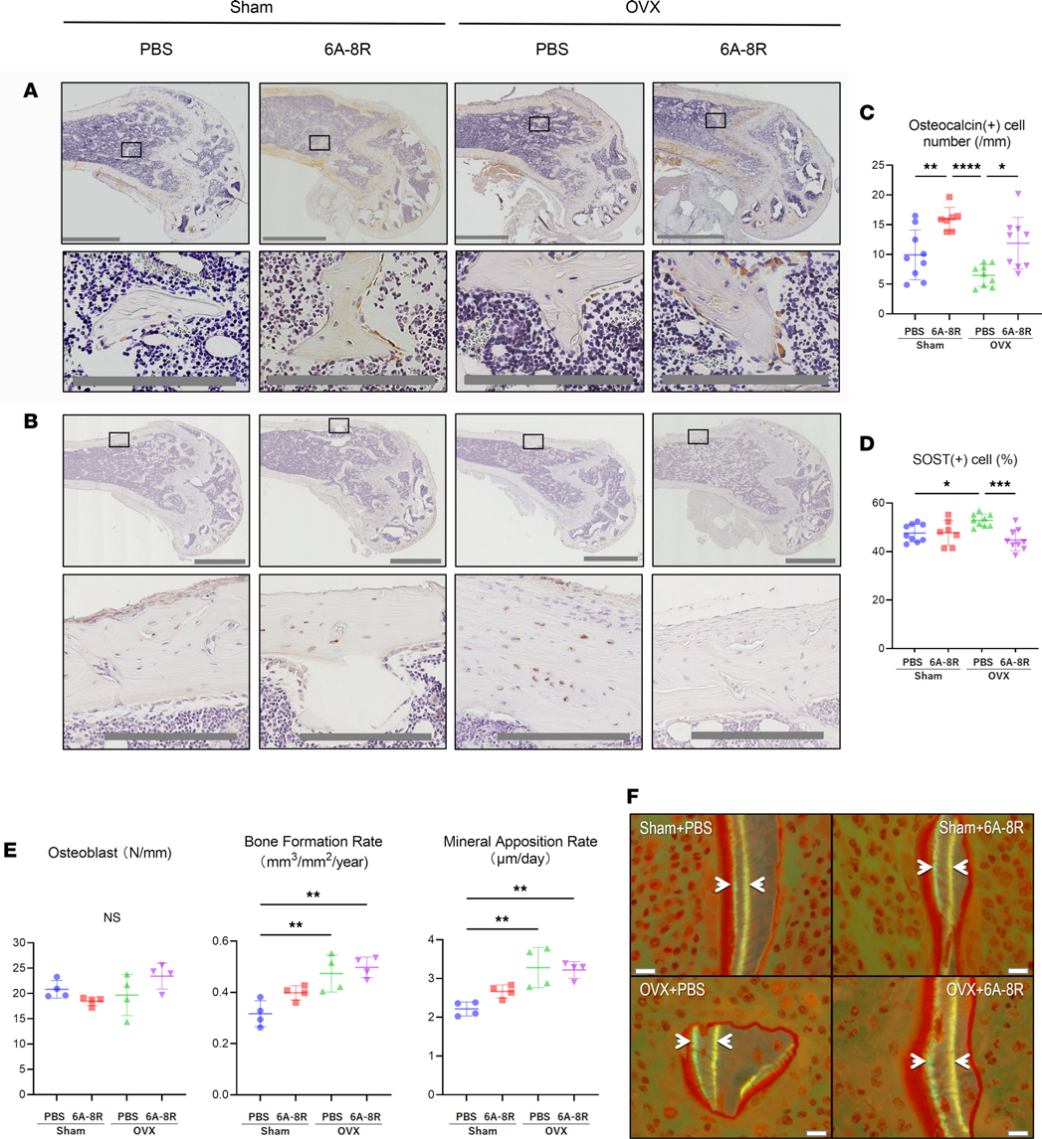
Histological and histomorphometric analysis of the distal part of the femur for osteoblasts and osteocytes in Sham-operated and OVX mice with or without intraperitoneal injection of 6A-8R (4 mg) 5 days per week for 4 weeks. (**A**) Osteocalcin staining. (**B**) Sclerostin staining. Scale bars (**A** and **B**): 1 mm (top rows) and 200 μm (bottom rows). (**C**) Plot of the number of osteocalcin-positive cells per unit trabecular surface. (**D**) Plot of the number of sclerostin-positive cells per total osteocytes. Data are expressed as mean ± SD (*n* = 7 or 9). (**E**) Plots of the number of osteoblasts (Ob.N) (N/mm), bone formation rate (BFR)/bone surface (BS) (mm³/mm²/year), and mineral apposition rate (MAR) (μm/day). (**F**) Images of MAR under fluorescent light (white arrow, double-labeled surface). Scale bars: 10 μm. Data are expressed as mean ± SD (*n* = 4) and were statistically analyzed using 1-way ANOVA and the Tukey-Kramer test. *P < 0.05; **P < 0.01; ***P < 0.001; ****P < 0.0001.

Figures 3 and 4 show the results of the histological examination of the distal part of the femur. The number of tartrate-resistant acid phosphatase–positive (TRAP-positive) cells was significantly higher in the OVX groups than in the Sham groups, according to the results of TRAP staining; however, the administration of 6A-8R significantly reduced the numbers of these cells (Figure 3, B and C).

Furthermore, in the Sham and OVX groups, 6A-8R administration significantly increased the number of osteocalcin-positive cells (Figure 4, A and C).

The number of sclerostin-positive cells in the cortical bone was significantly higher in the OVX groups than in the Sham groups; moreover, 6A-8R administration significantly reduced the number of sclerostin-positive cells in the OVX groups (Figure 4, B and D).

The results of the histomorphometric analysis are shown in Figure 3D and Figure 4E. The OVX groups had significantly higher numbers of osteoclasts (N.Oc)/bone surface (BS) and multinucleated osteoclasts (N.Mu.Oc)/BS as well as more eroded surface (ES)/BS than the Sham groups, and 6A-8R administration reduced these parameters in the OVX groups (Figure 3D).

Although there were no differences in the number of osteoblasts (N.Ob)/BS between groups, 6A-8R administration tended to increase both bone formation rate (BFR)/BS and mineral apposition rate (MAR) in the Sham groups (Figure 4, E and F).

Furthermore, cavities within paraffin-embedded tissue sections were designated as adipose tissue and quantified employing ImageJ software (version 1.52q, NIH). While a tendency toward augmentation was observed with OVX, and conversely, a decline was noted with 6A-8R treatment, these differences did not attain statistical significance (Supplemental Figure 5).

### Effects of 6A-8R on osteoclasts.

**Figure 5:**
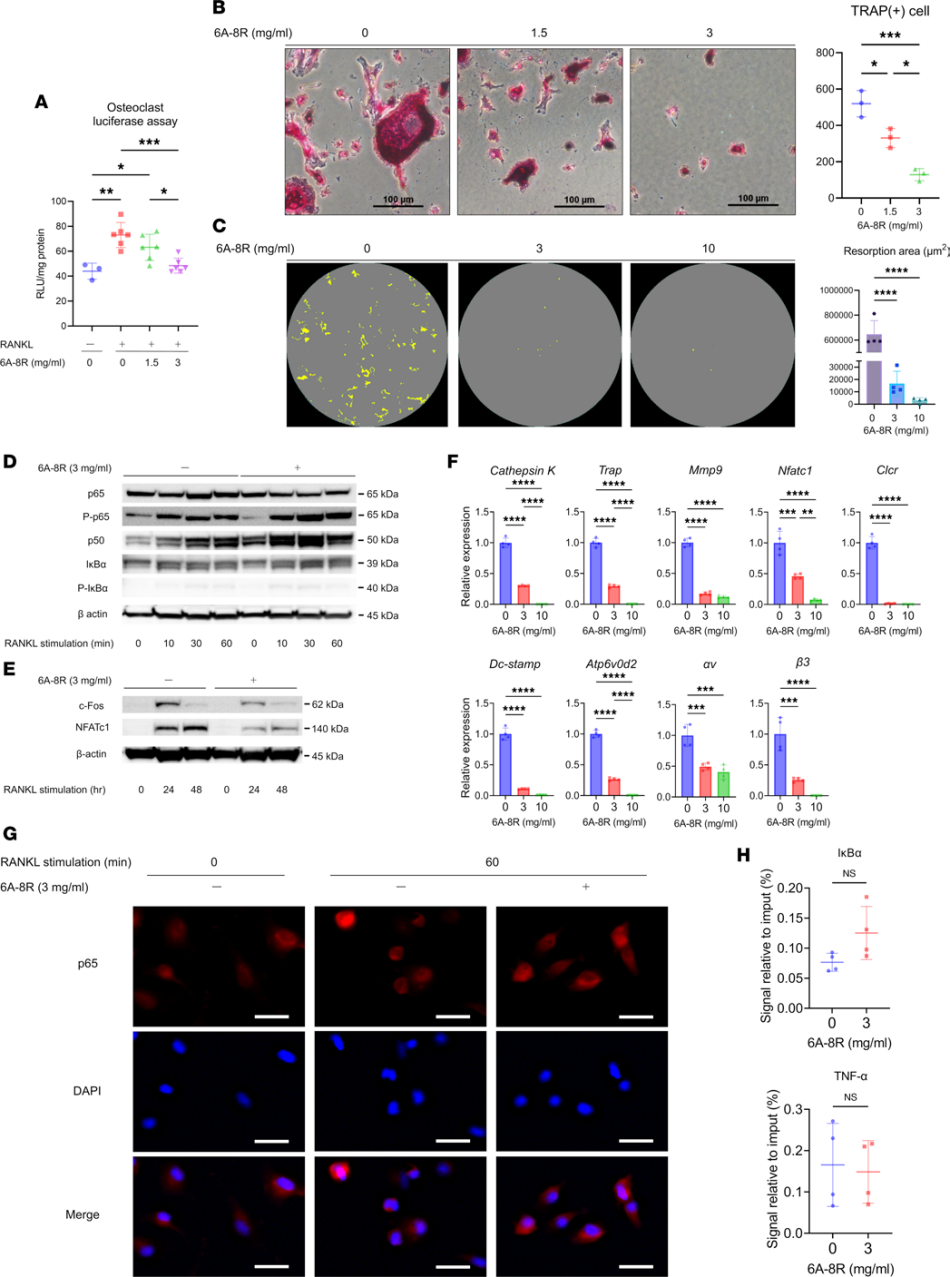
Effects of 6A-8R administration on osteoclasts. (**A**) Mouse bone marrow mononuclear cells (BMMCs) were transfected with a luciferase reporter gene (κB-Luc2P). After 24 hours of transfection, the cells were cultured with or without RANKL (50 ng/mL) for 6 hours together with indicated concentrations of 6A-8R. Data are expressed as mean ± SD (*n* = 3 or 6). (**B**) TRAP staining was performed, and the number of TRAP-positive cells was determined by microscopy. Scale bars: 100 μm. Data are expressed as mean ± SD (*n* = 3). (**C**) The bone resorption activity of osteoclasts was evaluated using an osteo-assay plate. Data are expressed as mean ± SD (*n* = 4). (**D** and **E**) Western blotting analysis of mouse BMMCs cultured with RANKL (50 ng/mL) with or without 6A-8R (3 mg/mL). (**F**) Changes in the expression of genes involved in osteoclast differentiation was assessed. Data are expressed as mean ± SD (*n* = 4). (**G**) Immunofluorescence microscopy analysis of p65 was performed on mouse BMMCs before and after stimulation with RANKL (50 ng/mL) and with or without 6A-8R (3 mg/mL). Red, p65 immunofluorescent staining; blue, 4′,6-diamidino-2-phenylindole (DAPI) nuclear staining. Scale bars: 20 μm. (**H**) CUT&RUN analysis of mouse BMMCs was performed 60 minutes after stimulation with RANKL (50 ng/mL) with or without 6A-8R (3 mg/mL). Data are expressed as mean ± SD (*n* = 4). *P < 0.05; **P < 0.01; ***P < 0.001; ****P < 0.0001 by 1-way ANOVA and the Tukey-Kramer test (**A**–**C** and **F**) or Mann-Whitney *U* test (**H**). NS, not significant.

To confirm the inhibitory effect of 6A-8R on NF-κB transcriptional activity in osteoclasts, we performed luciferase assays. Notably, 6A-8R (1.5 and 3.0 mg/mL) administration significantly reduced NF-κB transcription of osteoclasts induced by mouse BMMCs in a dose-dependent manner (Figure 5A).

Moreover, TRAP staining revealed that the administration of 6A-8R (1.5 and 3.0 mg/mL) significantly reduced RANKL-induced N.Mu.Oc in a dose-dependent manner (Figure 5B). Furthermore, a resorption pit assay revealed that the administration of 6A-8R (3.0 and 10 mg/mL) significantly suppressed RANKL-induced osteoclast resorption activity in a dose-dependent manner (Figure 5C).

Western blotting revealed that 6A-8R (3.0 mg/mL) promoted p65 phosphorylation (p-p65) and p50 induction in mouse BMMCs. However, there were no apparent changes in the amount or phosphorylation of IκBα (Figure 5D). In addition, 6A-8R inhibited the induction of c-Fos and NFATc1 (Figure 5E) and slightly inhibited the induction of p52 and RelB (Supplemental Figure 6). Given that p-p65/p50 is thought to exist in the nucleus without transcription of the target gene despite binding to DNA, it is plausible that expression of the NF-κB subunits is suppressed. Owing to the suppression of the turnover by negative feedback, the levels of expression of p-p65 and p50 increased. Notably, 6A-8R had no direct effect on IκBα, and there was no change in IκBα expression. The suppression of NF-κB by 6A-8R administration reduced the expression of c-Fos and NFATc1.

Figure 5F shows the results of quantitative real-time PCR of osteoclast-related mRNAs performed after 6A-8R (3.0 and 10 mg/mL) administration. The administration of 6A-8R significantly inhibited osteoclast-related mRNA (cathepsin K [*Ctsk*], *Trap*, *Mmp9*, *Nfatc1*, calcitonin receptor [*Calcr*], dendritic cell-specific transmembrane protein [*Dcstamp*], *Atp6v0d2*, integrin αv [*Itgav*], and β3 integrin [*Itgb3*]) expression in a dose-dependent manner. Furthermore, 6A-8R increased Fas-related mRNA (*Fas*, *Fasl*) expression and inhibited NF-κB–related mRNA (*RelB*, *Nfkb1*, *Nfkb2*) expression (Supplemental Figure 7). The effects of 6A-8R (3.0 mg/mL) on p65 nuclear translocation and on the interaction between p65 and DNA binding are shown in Figure 5, G and H.

Fluorescent immunostaining revealed that the administration of 6A-8R had no effect on the nuclear translocation of p65 (Figure 5G). Furthermore, evaluation of the association between p65 and IκBα or the TNF-α promoter region using CUT&RUN data analysis revealed that 6A-8R did not affect DNA binding of p65 (Figure 5H).

### Effects of 6A-8R on osteoblasts.

**Figure 6:**
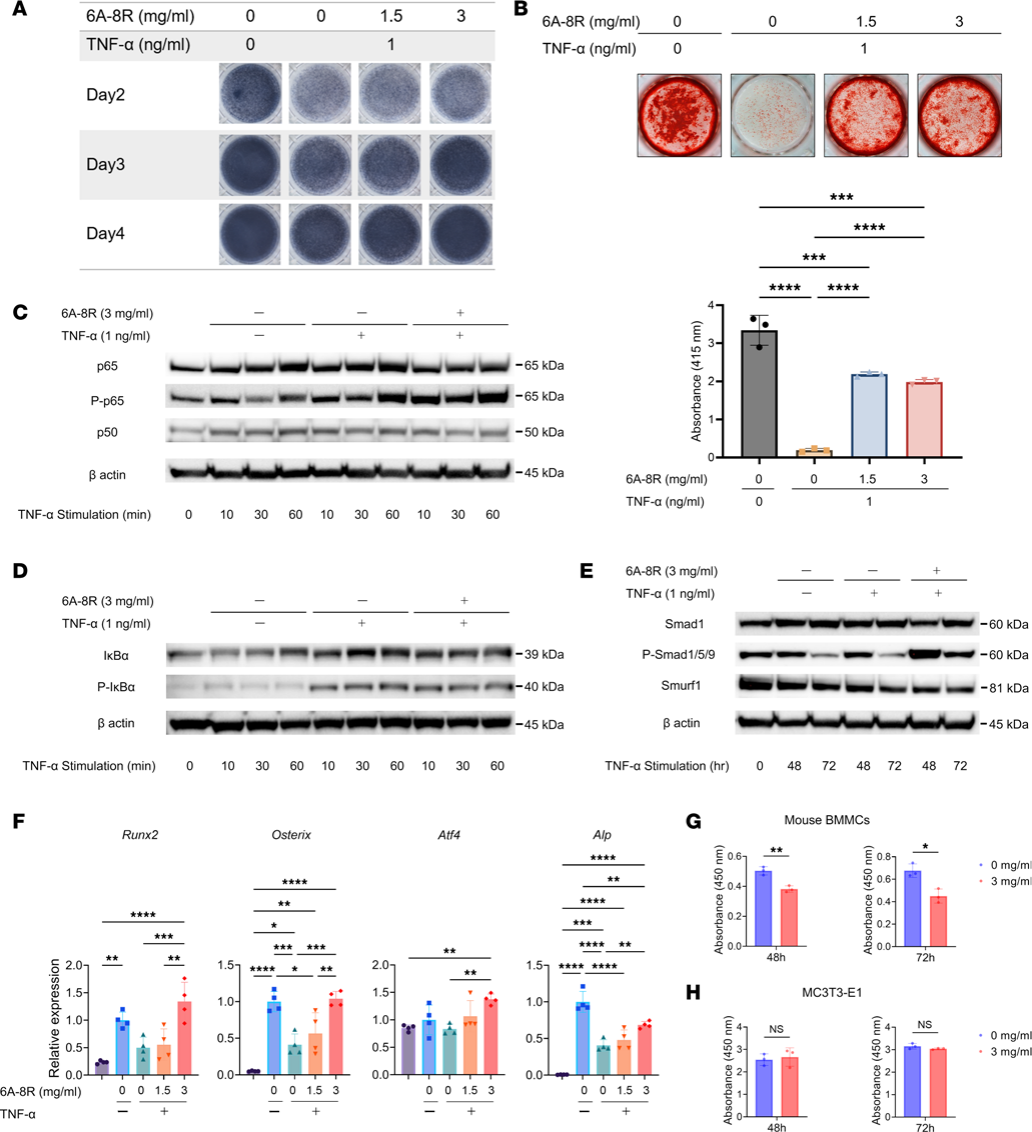
Effects of 6A-8R on osteoblasts. (**A**) The effect of 6A-8R on alkaline phosphatase (ALP) of MC3T3-E1 cells stimulated with TNF-α (1 ng/mL) was evaluated using ALP staining. (**B**) The effect of 6A-8R on the mineralization of MC3T3-E1 cells stimulated with TNF-α (1 ng/mL) was evaluated using Alizarin red staining. Data (bottom) are expressed as mean ± SD (*n* = 3). (**C**–**E**) The effects of 6A-8R on the phosphorylation of p65, expression of p50, phosphorylation of IκBα, phosphorylation of Smad1, and expression of Smurf1 after TNF-α stimulation were analyzed using Western blotting. (**F**) The effects of 6A-8R on the gene expression of runt-related transcription factor 2 (*Runx2*), *Osterix*, activating transcription factor 4 (*Atf4*), and *Alp* were analyzed using quantitative real-time PCR. Data are expressed as mean ± SD (*n* = 4). (**G** and **H**) The effects of 6A-8R on the proliferation of mouse bone marrow mononuclear cells (BMMCs) and MC3T3-E1 cells were evaluated using water-soluble tetrazolium assay. Data are expressed as mean ± SD (*n* = 3). *P < 0.05; **P < 0.01; ***P < 0.001; ****P < 0.0001 by 1-way ANOVA and the Tukey-Kramer test (**B** and **F**) or 2-tailed Student’s *t* test (**G** and **H**). NS, not significant.

As shown in Figure 6A, 6A-8R (1.5 and 3.0 mg/mL) increased alkaline phosphatase (ALP) staining in MC3T3-E1 cells in a dose-dependent manner. Mineralization was also evaluated in the same manner and showed significant improvement with 6A-8R administration (Figure 6B). When MC3T3-E1 cells were incubated with TNF-α (1.0 ng/mL) in the presence of recombinant human bone morphogenetic protein-2, 6A-8R (3.0 mg/mL) upregulated p-p65 similarly to that seen in osteoclasts (Figure 6C). In contrast, no changes in p50 expression were observed (Figure 6C). IκBα induction was marginally inhibited by 6A-8R (Figure 6D). Furthermore, Smurf1 induction was inhibited by 6A-8R (Figure 6E); therefore, p-Smad1/5/9 was upregulated (Figure 6E) (52). There were no obvious changes in the expression of Runx2, β-catenin, or JunB as a result of the administration of 6A-8R (Supplemental Figure 8).

According to quantitative real-time PCR, 6A-8R (1.5 and 3.0 mg/mL) inhibits the downregulation of the expression of *Runx2*, *Osterix*, and activating transcription factor 4 (*Atf4*) in a dose-dependent manner, (Figure 6F). Furthermore, it inhibits the downregulation of *Alp* gene expression in a dose-dependent manner, which is consistent with the results of ALP staining (Figure 6F).

### Effects of 6A-8R on the proliferation of mouse BMMCs and MC3T3-E1 cells.

According to in vitro cell proliferation analysis based on the water-soluble tetrazolium (WST) assay, 6A-8R (3.0 mg/mL) inhibited the proliferation of mouse BMMCs. In contrast, it did not inhibit the proliferation of MC3T3-E1 cells (Figure 6, G and H).

### Effects of 6A-8R on osteocytes.

**Figure 7:**
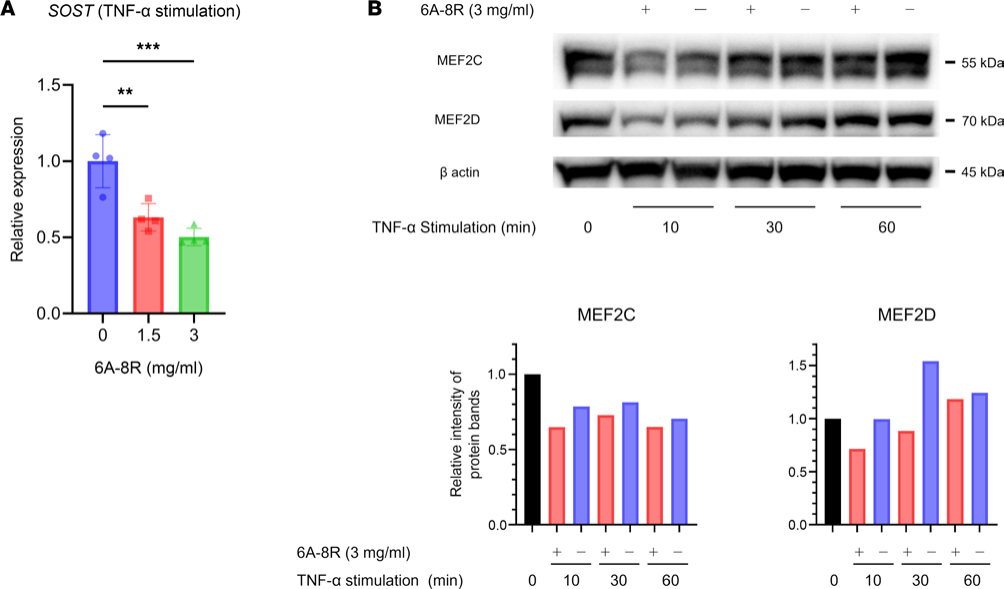
Effects of 6A-8R on osteocytes. (**A**) The expression of the sclerosteosis gene (*SOST*) in sarcoma osteogenic (SaOS-2) cells after TNF-α (1 ng/mL) stimulation with 6A-8R was analyzed using quantitative real-time PCR. Data are expressed as mean ± SD (*n* = 4) and were statistically analyzed using 1-way ANOVA and Tukey-Kramer test. **P < 0.01, ***P < 0.001. (**B**) Western blotting analysis of myocyte enhancer factor 2C (MEF2C) and MEF2D in SaOS-2 cells cultured with TNF-α (1 ng/mL) with or without 6A-8R (3 mg/mL). Volumetric analysis was performed utilizing β-actin as a loading control.

After stimulation with TNF-α (1.0 ng/mL), quantitative real-time PCR of sarcoma osteogenic (SaOS-2) cells revealed that 6A-8R (1.5 and 3.0 mg/mL) inhibited the expression of the sclerosteosis gene (*SOST*) (Figure 7A). Furthermore, when SaOS-2 cells were incubated with TNF-α (1.0 ng/mL), 6A-8R (3.0 mg/mL) suppressed the expression of myocyte enhancer factor 2C (MEF2C) and MEF2D (Figure 7B). Given that these factors act on the *SOST* enhancer region to enhance *SOST* expression, the above-mentioned findings may explain why 6A-8R has a suppressive effect on *SOST* expression (53). Sclerostin inhibits Wnt signaling, which leads to the inhibition of osteoblastogenesis and the promotion of osteoclastogenesis. Thus, 6A-8R administration can protect from degradative bone metabolism due to sclerostin.

## Discussion

In the present study, we have successfully devised a peptide, namely 6A-8R, with potent anti–NF-κB properties that effectively attenuates NF-κB transcriptional activity. Notably, this peptide demonstrated efficacy in mitigating OVX-induced osteoporosis in mice. In our previous study, administration of 40A-8R showed efficacy in a carrageenan-induced foot edema model, a croton oil–induced conjunctival inflammation model, a mite antigen–induced atopic dermatitis model, and a collagen-induced arthritis model, with no apparent adverse events (36). In addition, MTI-II and related peptides are considered relatively safe because MTI-II is ubiquitously expressed in various human tissues.

**Figure 8:**
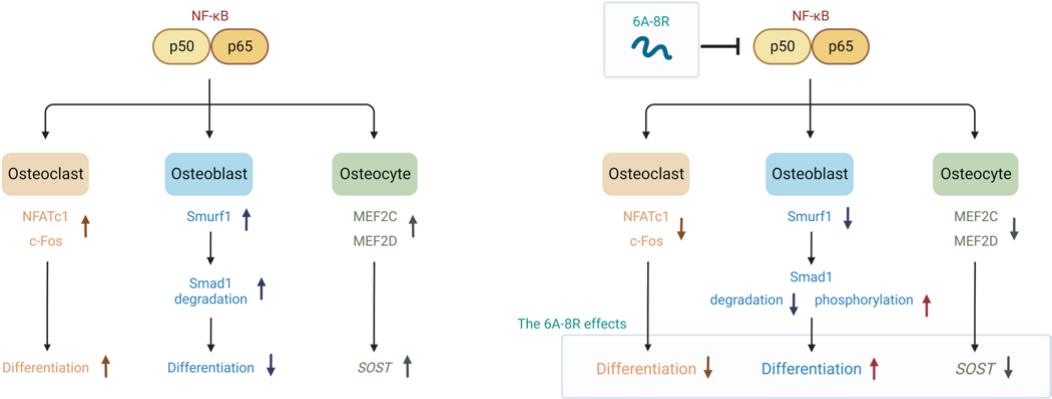
Hypothetical scheme summarizing the effects of NF-κB without (left) or with (right) 6A-8R on osteoclasts, osteoblasts, and osteocytes.

While the effectiveness of MTI-II and related peptides in combating osteoporosis has not been investigated, the present study substantiates their efficacy in mitigating this condition. As previously stated, 6A-8R significantly inhibits osteoclast differentiation; moreover, it shows positive effects, such as the promotion of osteoblast differentiation and inhibition of *SOST* expression in osteocytes. Figure 8 shows the assumed mechanisms of 6A-8R in bone metabolism. For example, systemic administration of 6A-8R improves osteoporosis in OVX mice by inhibiting osteoclastogenesis and sclerostin expression and inducing osteocalcin expression without apparent side effects. It has been reported that NF-κB is important in RANKL-induced osteoclastogenesis via both canonical and alternative pathways (54–57). The NF-κB subunit p50 primarily participates in the canonical pathway with p65, whereas the NF-κB subunit p52 participates in the alternative pathway with RelB. A previous study found that knockout mice that lack p50 or p52 develop osteopetrosis in vivo (58). Furthermore, it has been reported that the downregulation of p50 or p52 inhibits osteoclast differentiation to some extent, whereas double knockout of the 2 factors completely inhibits osteoclast differentiation in vitro (59).

Previous studies have reported that NEMO-binding-domain peptides with inhibitory effects upstream of the NF-κB signaling cascade are effective in animal models of osteoporosis; however, none of these peptides have been used in clinical settings (24, 60–62). The lack of clinical use of these peptides may be attributed to the adverse events caused by inhibition upstream of the NF-κB signaling cascade (24).

Following TNF-α stimulation, MTI-II inhibited NF-κB transcriptional activity by directly binding to NF-κB (36).

In this study, we showed that 6A-8R effectively inhibits the transcriptional activity of NF-κB, thereby inhibiting osteoclast differentiation via the inhibition of the downstream cascade in the transcriptional activity of NF-κB, which is similar to the effect of MTI-II. Moreover, 6A-8R significantly decreased osteoclast number and bone erosion in vivo, which is consistent with in vitro findings.

NF-κB inhibits osteoblast proliferation and differentiation (16–19) by inducing Smurf1 activation, leading to Smad (17, 21, 22), Runx2, and β-catenin degradation (20–23). In the present study, we found that 6A-8R decreases Smurf1 expression and increases Smad phosphorylation (52), promoting osteoblast differentiation (MC3T3-E1 cells).

We also found that 6A-8R promotes osteoblast differentiation (osteocalcin expression) in vivo. However, 6A-8R administration did not significantly increase BFR or MAR levels in vivo, but the effect of 6A-8R may have been blunted by decreased total bone turnover.

Previous studies have linked OVX to increased TNF-α in T cells and increased sclerostin expression in osteocytes (40–51). Notably, sclerostin inhibits Wnt signaling, which in turn inhibits osteoblast differentiation and promotes osteoclastogenesis by inhibiting osteoprotegerin production (63). In the present study, we confirmed that OVX induces sclerostin expression and that 6A-8R significantly reduces the number of sclerostin-positive osteocytes in vivo. We also found that 6A-8R reduced *SOST* expression in SaOS-2 cells. This finding may be due to suppressed expression of MEF2C and MEF2D, which are critical to *SOST* expression (53).

In summary, 6A-8R administration inhibited osteoclastogenesis and sclerostin expression but promoted osteoblastogenesis to a limited extent in vivo. The positive effect of 6A-8R on osteoblasts was not apparent in vivo, probably because of the inhibition of both osteoclastogenesis and total bone turnover by 6A-8R.

This study has some limitations. First, the study was conducted in relatively young 8-week-old mice. Second, 6A-8R was administered only via intraperitoneal injection at a specific dose, frequency, and duration based on previous studies in the relevant literature. Regarding its clinical application, further research is necessary to optimize dosage and route of administration and for long-term safety for larger animals. Third, the effects of 6A-8R on immune cells are not known and should be clarified in future studies. Lastly, the potential for further investigation into the pharmacokinetics of 6A-8R remains, encompassing essential aspects like its half-life.

Our results indicate that this anti–NF-κB peptide may be considered an uncoupling antiosteoporotic agent that inhibits osteoclastogenesis and sclerostin expression.

## Methods

### Animals and protocols

Seven-week-old female C57BL/6J mice were purchased from Charles River Laboratories. After 1 week of acclimatization, the mice were anesthetized with intraperitoneal injections of 0.3 mg/kg medetomidine (Nippon Zenyaku Kogyo Co. Ltd.), 4.0 mg/kg midazolam (Astellas Pharma Inc.), and 5.0 mg/kg butorphanol (Meiji Seika Pharma Co. Ltd.). Subsequently, Sham or OVX with dorsal skin incision was performed, as described in a previous study (64). According to our previous study, PBS (250 μL) or 6A-8R dissolved in PBS (16 mg/mL, 250 μL) was administered by intraperitoneal injection 5 times per week for 4 weeks from postoperative day 1 (65). The mice were divided into the following 4 groups: Sham + PBS, Sham + 6A-8R, OVX + PBS, and OVX + 6A-8R. All the mice were housed in a temperature- and humidity-controlled facility with a 12-hour light/dark cycle, and they had free access to standard chow and water for 4 weeks before being euthanized (Figure 2A).

We developed 6A-8R as described above, and the peptide was purchased from Biomatik. In our previous study (36), we confirmed the antiinflammatory effects of intraperitoneally administered 40A-8R (3.5 mg) in rats with collagen-induced arthritis; moreover, the antiinflammatory effects on HeLa cells (DS Pharma Biomedical) were confirmed by luciferase assay with addition of 40A-8R (0.5 mg/mL). Compared with 40A-8R, 3.0 mg/mL 6A-8R was required for the equivalent inhibition of NF-κB (Figure 1D). Thus, we decided to administer 4.0 mg of 6A-8R into mice based on the mice’s body weight.

### High-resolution micro-CT analysis

The distal part of the right femur of the mice (1000-μm width above the growth plate) was evaluated using high-resolution micro-CT (SkyScan 1272; Bruker Corporation) with voltage at 90 kV and current at 160 mA.

The results were analyzed using CTAn software (Bruker Corporation) for parameters such as BV, TV, BMD, Tb.Th, Tb.N, Tb.Sp, Tt.Ar, and Ct.Ar, as described in previous studies (65, 66).

### Histology and immunohistochemistry

The bones were fixed with 4% paraformaldehyde in PBS and decalcified with 20% EDTA. The bones were then dehydrated by incubation in an ethanol series and embedded in paraffin wax before being sliced into 3-μm-thick sections. The right femurs were cut along the sagittal axis, and the sections were stained with hematoxylin and eosin, Safranin O–fast green, and TRAP substrate (Cosmo Bio) according to standard protocols. The number of TRAP-positive cells per trabecular surface in the distal part of the right femurs (1,000 μm width above the growth plate) was determined. In addition, osteocalcin (Takara Bio, MK127) and sclerostin (R&D Systems, AF1589) staining was performed according to the manufacturer’s protocol. The sections was then incubated with the secondary antibody (VECTASTAIN ABC Rabbit IgG Kit, PK-4005, Vector Laboratories) and stained with 3,3′-diaminobenzidine tetrahydrochloride (Nichirei Biosciences) according to the manufacturer’s protocol.

### Histomorphometrical analysis

Overall, 5 and 2 days before being euthanized, all the mice were injected with 20 mg/kg tetracycline (Sigma-Aldrich) and 10 mg/kg calcein (Sigma-Aldrich) to label active bone formation, as described in a previous study (65). The following histomorphometric parameters were measured: N.Oc/BS, N.Mu.Oc/BS, ES/BS, N.Ob/BS, BFR/BS, and MAR. Standard bone histomorphometric nomenclature, symbols, and units were used as described in the report of the American Society for Bone and Mineral Research Histomorphometry Nomenclature Committee (67). Osteoclasts and osteoblasts were captured in Villanueva bone stain under high magnification (×800) using natural light. Osteoclasts exhibit adherence to the eroded surface, and their cytoplasm displays a milky white appearance under fluorescence microscopy. Moreover, these cells possess distinctive characteristics, including prominent nuclei and the accumulation of chromatin along the periphery and within the central region. Osteoblasts, on the other hand, were recognized by their alignment along the osteoid, accompanied by blue cytoplasm and deeply stained nuclei.

### Side effects of 6A-8R in the liver and kidneys

Two specimens from each group were sent to KAC Co. Ltd. in order to confirm the side effects of 6A-8R treatment, such as atrophy and scarring, in the liver and kidneys.

### Cell proliferation assay

Mouse BMMCs and MC3T3-E1 cells (Riken Cell Bank) were cultured in 96-well plates at concentrations of 2.0 × 10^4^ cells per well and 5.0 × 10^3^ cells per well, respectively, in α minimum essential medium (αMEM) (Nacalai Tesque) containing 10% fetal bovine serum (FBS) (Equitech-Bio) and 1% penicillin and streptomycin (Sigma-Aldrich). The cells were incubated for 1 day, after which the medium was supplemented with 6A-8R (3.0 mg/mL) for 3 days. Cell viability was evaluated using the WST assay kit (Dojindo Laboratories) according to the manufacturer’s protocol.

### Luciferase assay

#### Transfection of HeLa cells with 12A-6R and 6A-6R genes.

HeLa cells were transfected with 60 ng per well of the κB-Luc2P vector (pGL4.32; Promega), 20 ng per well of the TK-hRLuc vector (pGL4.74; Promega), and 120 ng per well of 12A-6R or 6A-6R expression plasmid vector (pTriEx-4; Novagen, Merck KGaA). In 48-well white plates, the cells were grown to 80% confluence before being transfected the next day with FuGENE HD (Promega) according to the manufacturer’s protocol. TNF-α (1.0 ng/mL) was added to the cells 44 hours after transfection. A dual-luciferase assay system was used to detect luciferase activity after 4.5 hours of incubation (Promega).

#### Treatment with 12A-8R and 6A-8R in HeLa cells.

As previously described, HeLa cells were transfected with 60 ng per well of the κB-Luc2P vector (pGL4.32; Promega) and 20 ng per well of the TK-hRLuc vector (pGL4.74; Promega). The cells were incubated with 12A-8R or 6A-8R (1.5 and 3.0 mg/mL) after 10 hours of transfection. TNF-α (1.0 ng/mL) was added to the cells 38 hours after incubation. The luciferase activity was detected after 4.5 hours of incubation.

#### 6A-8R treatment in mouse BMMCs.

Mouse BMMCs were transfected with κB-Luc2P vector (pGL4.32; Promega). The cells were grown to 80% confluence in 96-well white plates and were transfected the next day using FuGENE 6 (Promega) according to the manufacturer’s protocol.

After 24 hours of transfection, the cells were incubated with or without 6A-8R (1.5 and 3.0 mg/mL). After 2 hours of incubation, the cells were stimulated with RANKL (50 ng/mL). After 24 hours of incubation, Steady-Glo reagent (Promega) was added to each well, and a waiting period of 5 or more minutes was applied. Luminescence was measured using a Centro XS3 LB960 luminometer (Berthold).

### In vitro osteoclastic differentiation assay

Primary osteoclasts were obtained from BMMCs flushed from the femurs and tibiae of 8-week-old male C57BL/6J mice. These cells were cultured overnight at 37°C in α-MEM containing 10% FBS and 1% penicillin and streptomycin with 10 ng/mL M-CSF (R&D Systems), as described in a previous study (68). Nonadherent cells were seeded in 12-well and 96-well plates at 1.5 × 10^5^ cells per well and 1.0 × 10^5^ cells per well, respectively, in the above-mentioned medium. The cells were stimulated with 10 ng/mL M-CSF and 50 ng/mL RANKL (R&D Systems) to induce osteoclastogenesis and incubated with or without 6A-8R (3.0 mg/mL) for 5 days, as described in a previous study (68). The medium was changed every 2 days.

### In vitro TRAP staining of osteoclastic cells

Mouse BMMCs were incubated for 5 days in 96-well plates as described above. At the end of the incubation period, the cells were fixed with 10% formalin and washed with PBS. TRAP staining was performed using a TRAP staining kit (Cosmo Bio) according to the manufacturer’s protocol. The total number of TRAP-positive cells with 3 or more nuclei was determined as described in a previous study (68).

### In vitro resorption pit assay of osteoclastic cells

As described above, mouse BMMCs were incubated for 5 days using Osteo-Assay Surface 96-Well Multiple Well Plates (Corning). To analyze the surface of wells for pit formation, the medium was aspirated from the wells, and 100 μL of 6% sodium hypochlorite (bleach solution) was added. The cells were incubated with a bleach solution at room temperature for 5 minutes, after which the wells were washed twice with distilled water and allowed to dry at room temperature for 3–5 hours. Individual pits or multiple pit clusters were observed using a fluorescence microscope (BZ-X800, Keyence), as described in a previous study (68).

### Quantitative real-time PCR assay

Total RNA was extracted from cells in a 12-well plate using an RNeasy Mini Kit (Qiagen) and was subsequently converted to complementary DNA using ReverTra Ace qPCR RT Master Mix with gDNA Remover (Toyobo). Gene expression was measured using quantitative real-time PCR with SYBR Green Master Mix (Thermo Fisher Scientific) in a Step One Plus Real-Time PCR System (Thermo Fisher Scientific). The primer sequences used for quantitative real-time PCR are listed in Supplemental Table 1. mRNA levels were normalized to the hypoxanthine phosphoribosyltransferase 1 (*HPRT1*) or glyceraldehyde-3-phosphate dehydrogenase (*GAPDH*) level and then calculated using the ΔΔCT method.

### Western blotting

Cells were homogenized with 100 μL of radioimmunoprecipitation assay buffer (Thermo Fisher Scientific), and complete cell lysis was performed for 10 minutes on ice using a sonicator. The lysates were centrifuged at 12,000 rpm for 5 minutes at 4°C to remove debris, and the supernatants were used for electrophoresis after a protein assay using a bicinchoninic acid assay kit (Thermo Fisher Scientific) and transferred to a polyvinylidene difluoride membrane (Nippon Genetics). The amounts of protein were 45 μg for osteoclasts, 30 μg for MC3T3-E1 cells, and 50 μg for SaOS-2 cells (Riken Cell Bank). Western blotting was performed using the following antibodies purchased from Cell Signaling Technology: anti–c-Fos (catalog 2250), anti-NFATc1 (catalog 8032), anti–p-p65 (Ser536) (catalog 3031), anti-p65 (catalog 8242), anti-p105/p50 (catalog 13586), anti-p100/p52 (catalog 4882), anti-RelB (catalog 10544), anti–p-IκBα (Ser32) (catalog 2859), anti-IκBα (catalog 4812), anti-Runx2 (catalog 12556), anti–β-catenin (catalog 8480), anti-JunB (catalog 3753), anti-Smurf1 (catalog 2174), anti–p-Smad1 (S463/465)/Smad5 (S463/465)/Smad9 (S465/467) (catalog 13820), anti-Smad1 (catalog 6944), anti-MEF2C (catalog 5030), anti-MEF2D (catalog 56830), and anti–β-actin (catalog 4967). Each antibody was diluted 1:000. In addition, we purchased anti-COX2 (ab52237; 1:1000) and anti-GAPDH (ab9485, 1:2500) antibodies from Abcam. Membranes were sequentially probed with multiple antibodies by washing 15 minutes in WB Stripping Solution Strong (Nacalai Tesque) at room temperature, between each experiment.

### Evaluation of p65 nuclear translocation and interaction of p65 and DNA

The effect of 6A-8R (3.0 mg/mL) on p65 nuclear translocation was confirmed by immunofluorescence (Cell Signaling Technology) according to the manufacturer’s protocol. The binding of p65 to the IκBα or TNF-α promoter region was also measured using the CUT&RUN method (Cell Signaling Technology) according to the manufacturer’s protocol. The primer sequences used for CUT&RUN are listed in Supplemental Table 1.

### In vitro osteogenic differentiation assay

MC3T3-E1 cells were cultured in α-MEM containing 10% FBS and 1% penicillin and streptomycin at 37°C. The cells were cultured in 12-, 24-, and 96-well plates at 1.5 × 10^5^ cells per well, 5.0 × 10^4^ cells per well, and 5.0 × 10^3^ cells per well, respectively, in the above-mentioned medium and stimulated with 10 mM β-glycerophosphate (Calbiochem) and 50 μg/mL ascorbic acid (Sigma-Aldrich) to induce osteoblastogenesis. Quantitative real-time PCR was performed on day 3.

SaOS-2 cells of the osteocytic cell lineage were cultured in Dulbecco’s modified Eagle medium (Nacalai Tesque) containing 10% FBS and 1% penicillin and streptomycin at 37°C. The cells were cultured in 6-well and 24-well plates at 1.0 × 10^6^ cells per well and 1.0 × 10^5^ cells per well, respectively, in the above-mentioned medium and stimulated with 5 mM β-glycerophosphate and 50 μg/mL ascorbic acid to induce osteocytogenesis.

### ALP staining

MC3T3-E1 cells were treated with recombinant human bone morphogenetic protein-2 (100 ng/mL) with or without TNF-α (1.0 ng/mL) and 6A-8R (1.5 and 3.0 mg/mL) after the cells reached confluence and were incubated for 3 days.

After fixation with 10% formalin, the cells were washed twice with PBS, and ALP staining was performed using BCIP/NBT Color Development Substrate (Promega) incubated for 20 minutes.

### Alizarin red staining

MC3T3-E1 cells were treated with recombinant human bone morphogenetic protein-2 (100 ng/mL) with or without TNF-α (1.0 ng/mL) and 6A-8R (1.5 and 3.0 mg/mL) after the cells reached confluence and were incubated for 20 days. The medium was changed every 2 days. After fixation with 10% formalin, the cells were washed twice with distilled water, and Alizarin red staining was performed using 1% Alizarin red solution (MUTO PURE CHEMICALS) incubated for 15 minutes. Then, the cells were washed twice with distilled water, the dye was eluted using 5% formic acid (FUJIFILM Wako Pure Chemicals), and the absorbance was measured at 415 nm.

### Statistics

All data are expressed as mean ± SD. One-way analysis of variance (ANOVA) followed by Tukey-Kramer post hoc test and Mann-Whitney *U* test were used to analyze all parameters between groups. Statistical analyses were performed using GraphPad Prism 9. Statistical significance was set at *P* less than 0.05.

### Study approval

Before the study was started, all experimental protocols were approved by the Ethics Review Committee for Animal Experimentation of Osaka University School of Medicine. The study approval number is 02-257-004.

### Data availability

All data in the manuscript and supplemental material presented in figures are provided in the Supporting Data Values file. Any additional information required to reanalyze the data reported in this paper is available from the corresponding author upon a reasonable request.

## Author contributions

KT and KE take responsibility for the integrity of the work as a whole, from inception to the completed manuscript. KT, KO, YE, MH, and KE conceived and designed the study. KT, KO, and KE analyzed and interpreted data and contributed statistical expertise. KT, KO, AM, GO, AG, TM, T Kurihara, YF, T Kanamoto, KN, SO, and KE provided administrative, technical, or logistic support. KT, KO, and KE wrote the manuscript. KT, KO, TM, and KE conducted the experiments. MH, T Kanamoto, KN, SO, and KE supervised the study.

## Supplementary Material

Supplemental data

Supporting data values

## Version 1 11/22/2023

Electronic publication
