# Supplementary material for: Anti–NF-κB peptide derived from nuclear acidic protein attenuates ovariectomy-induced osteoporosis in mice
Source: JCI Insight. 2023 Nov 22;8(22):e171962. doi: 10.1172/jci.insight.171962 (PMC10721323; doi:10.1172/jci.insight.171962)
Supplement: Supplemental data [file jciinsight-8-171962-s172.pdf]

Supplemental Figures

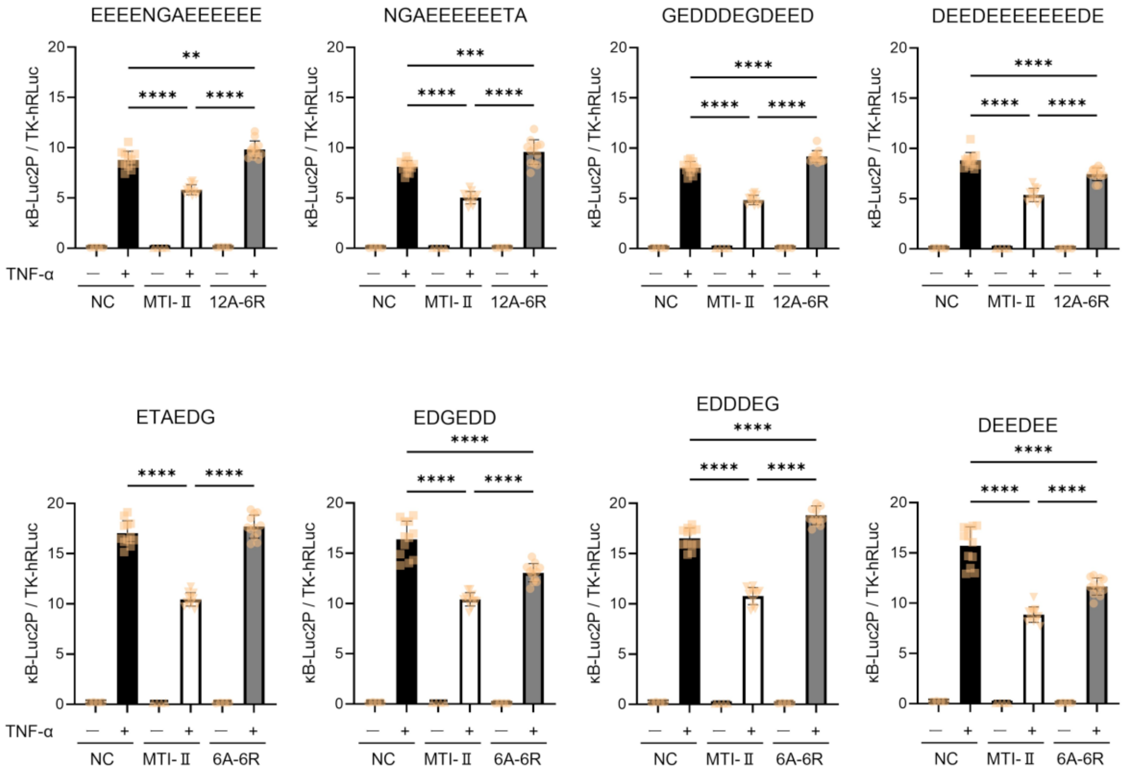

Supplemental Figure 1. Nuclear factor-kappa B (NF-κB)-induced luciferase activity was measured in Henrietta Lacks (HeLa) cells transfected with macromolecular translocation inhibitor II (MTI-II), 12A-6R, and 6A-6R expression vectors along with two luciferase reporter genes (κB-Luc2P and TK-hRLuc). Results for the remaining 12A-6R and 6A-6R candidates (not boxed in Figure 1C), which were less effective in suppressing NF-κB, are shown individually. Luciferase activity was measured 4.5 h after stimulation with tumor necrosis factor-α (TNF-α) (1 ng/mL). Data are expressed as a ratio of κB-Luc2P activity to TK-hRLuc activity (internal control) and are presented as mean ± standard deviation (SD) (n = 4, without TNF-α or n = 12, with TNF-α).

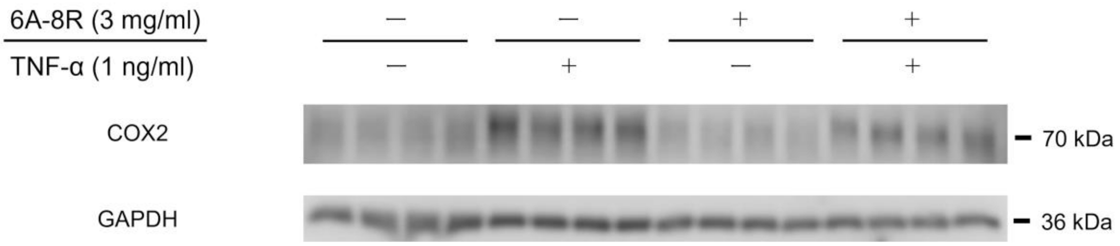

Supplemental Figure 2. Western blotting analysis of the effects of 6A-8R (3 mg/mL) on protein expressions of cyclooxygenase 2 (COX2) and glyceraldehyde-3-phosphate dehydrogenase (GAPDH) in Henrietta Lacks (HeLa) cells 4.5 h after stimulation with tumor necrosis factor-α (TNF-α).

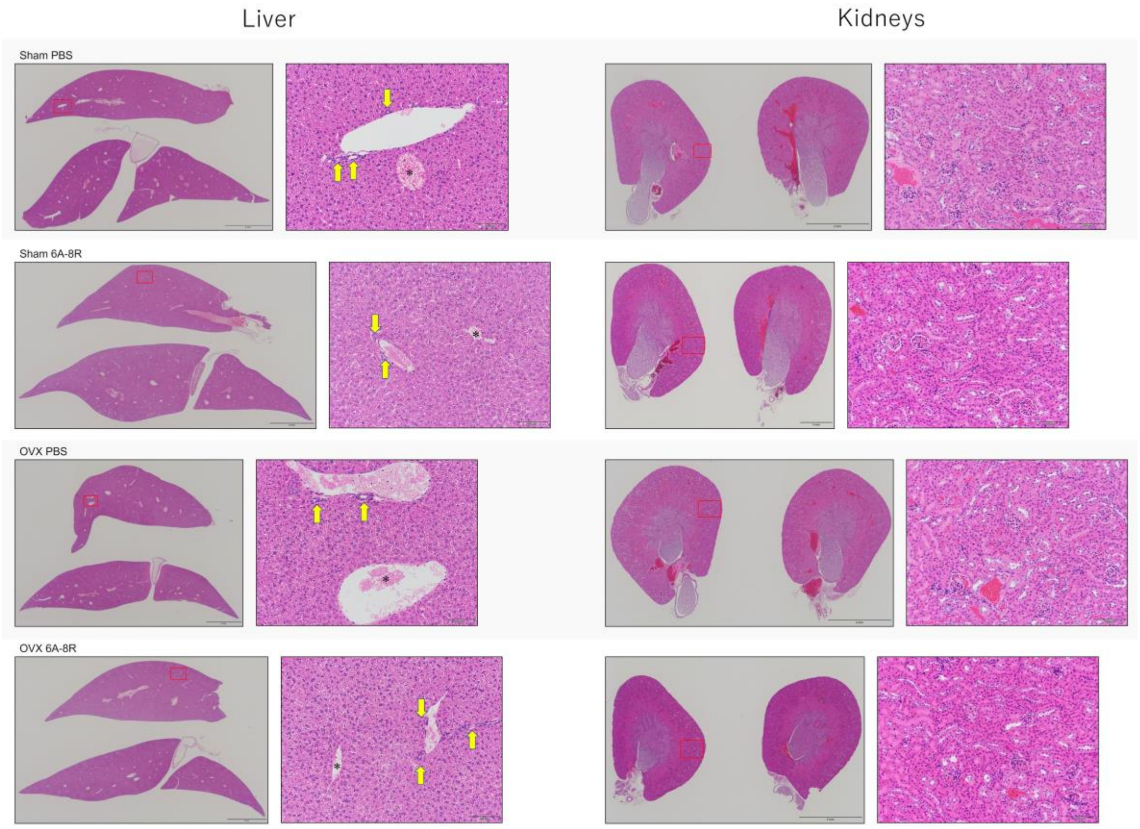

Supplemental Figure 3. Microscopic images of the liver and kidney sections from mice of the Sham + phosphate-buffered saline (PBS), Sham + 6A-8R, ovariectomized (OVX) + PBS, and OVX + 6A-8R groups. The injection dose and schedule are indicated in Figure 2A. The yellow arrows and blue asterisks indicate the central vein and interlobular bile duct, respectively (bar: 2 mm or 100 μm for whole or magnified view, respectively).

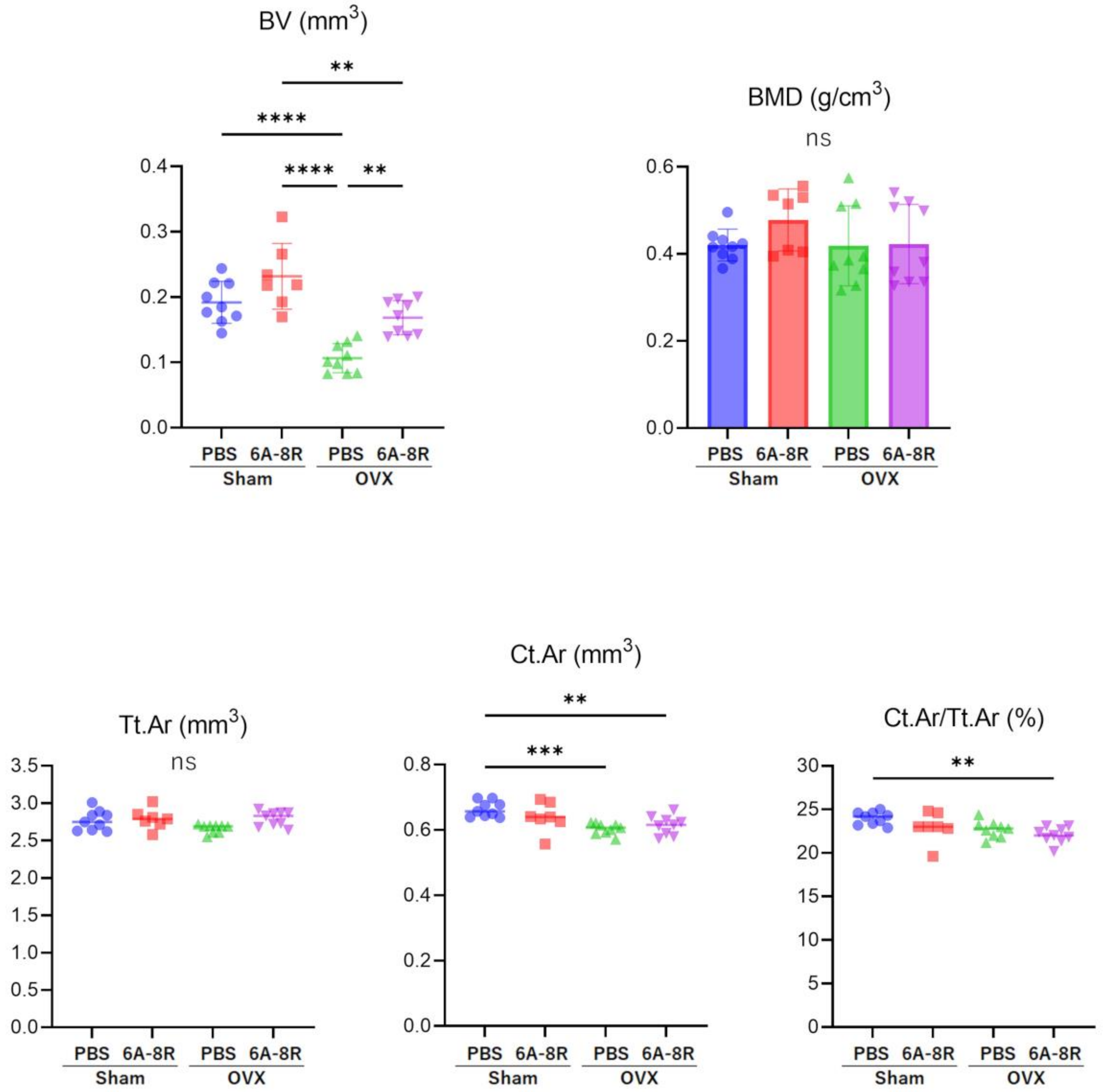

Supplemental Figure 4. Effects of 6A-8R on ovariectomized (OVX) mice. Cancellous bone volume (BV), bone mineral density (BMD), total area (Tt.Ar), cortical area (Ct.Ar), and Ct.Ar/Tt.Ar. Data are expressed as mean ± SD (n = 7 or n = 9). Data were statistically analyzed using one-way ANOVA and Tukey–Kramer test (**$p < 0.01$, ***$p < 0.001$, ****$p < 0.0001$). ns, not significant.

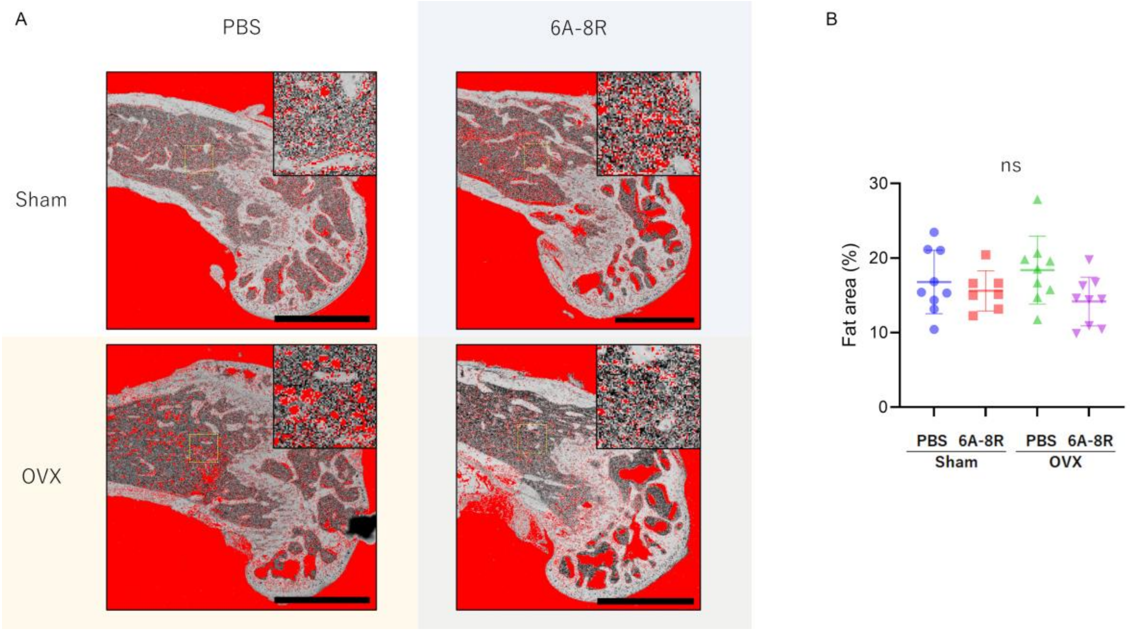

Supplemental Figure 5. Quantification of the fat marrow in the distal part of the femur in Sham-operated and ovariectomized mice with or without intraperitoneal injection of 6A-8R (4 mg) 5 days per week for 4 weeks. (**A**) Comparison of fat marrow area in the distal part of the femur in histological sections. A 300×300-μm2 region of interest was extracted from the secondary spongiosa (bar: 1 mm). The fat marrow area (red) was color-coded with ImageJ software (version 1.52q, U. S. National Institutes of Health). (B) The percentage of fat marrow area in each group. Data are expressed as mean ± SD (n = 7 or n = 9). Data were statistically analyzed using one-way ANOVA. ns, not significant.

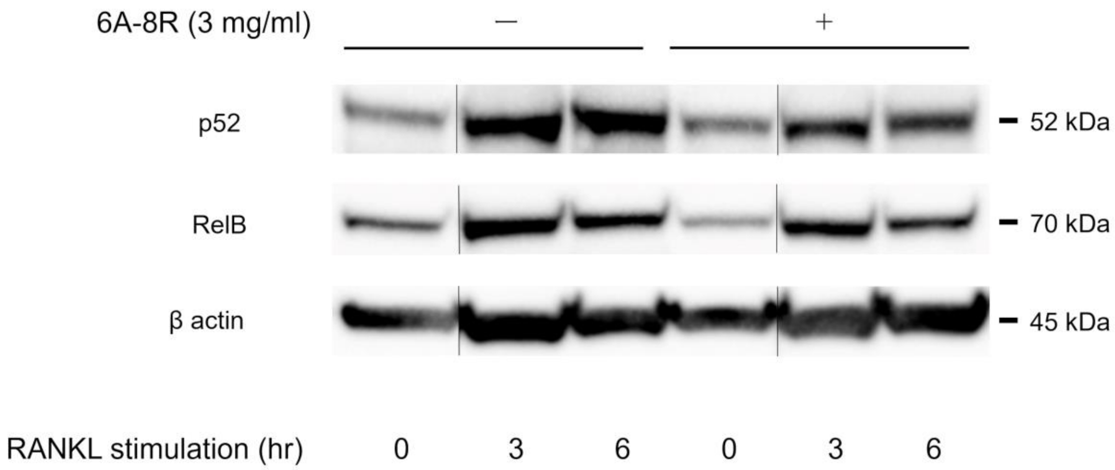

Supplemental Figure 6. Western blotting analysis of mouse bone marrow mononuclear cells after stimulation with receptor activator of NF-κB ligand (RANKL) (50 ng/mL) with or without 6A-8R (3 mg/mL).

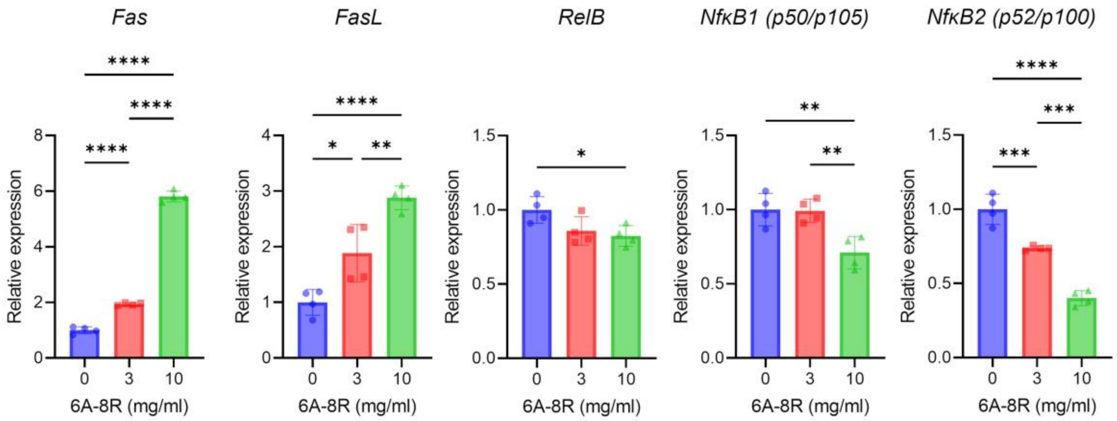

Supplemental Figure 7. Changes in the expressions of genes involved in the apoptosis and the nuclear factor-kappa B subunit genes of mouse bone marrow mononuclear cells were evaluated. Data are expressed as mean ± SD (n = 4). Further, data were statistically analyzed using one-way ANOVA and Tukey–Kramer test (*p < 0.05, **p < 0.01, ***p < 0.001, ****p < 0.0001).

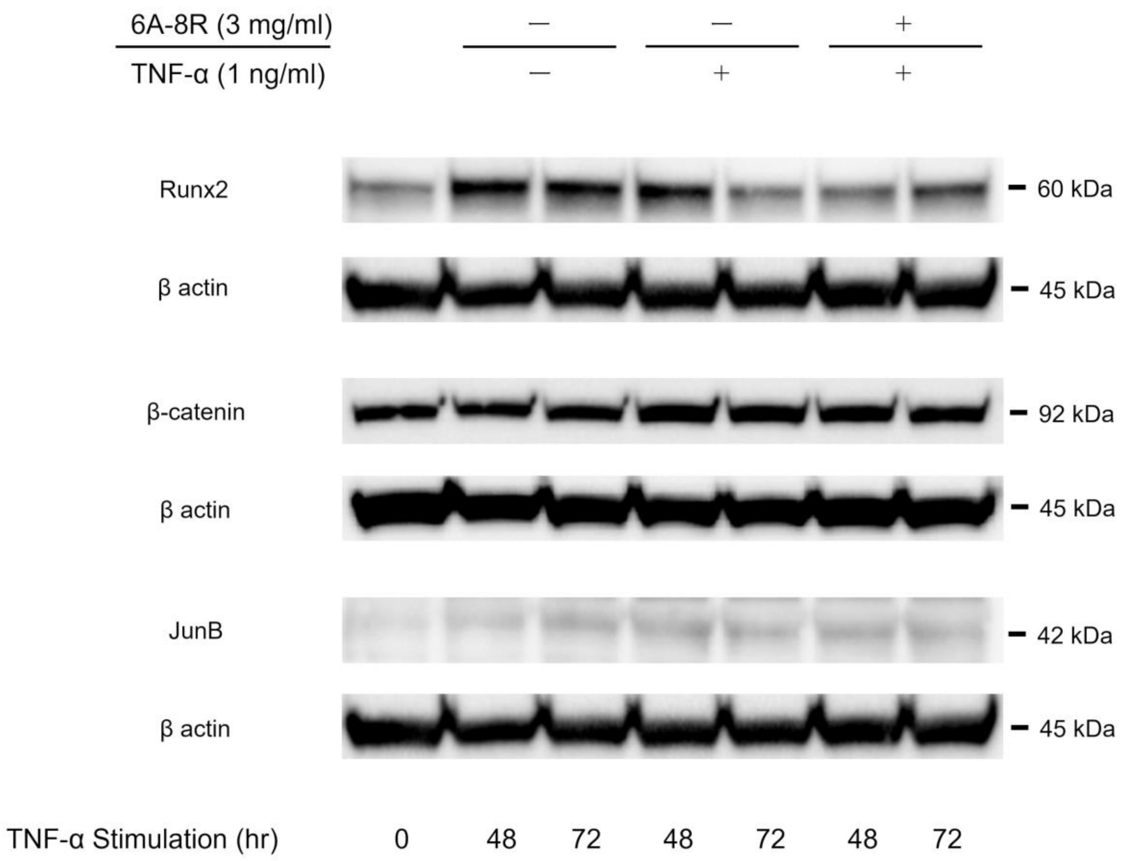

Supplemental Figure 8. Western blotting analysis of MC3T3-E1 cells after stimulation with tumor necrosis factor-α (TNF-α) (1 ng/mL) with or without 6A-8R (3 mg/mL) in the presence of recombinant human bone morphogenetic protein-2 (100 ng/mL).

Supplemental Table

Supplemental Table 1 Primers used in RT-PCR and CUT&RUN

Genes	Forward (5′ –3′)	Reverse (5′ –3′)
MTI-Ⅱ	ATGTCGGAGAAGAGCGTGGAG	CCGACGCCCCGTTTTCTG
Cathepsin K	CCATATGTGGGCCCAGGATG	TCAGGGCTTTCTCGTTCCC
TRAP	GGGACAATTTCTACTTCACTGGAG	TCAGAGAACACGTCCTCAAAGG
MMP9	CCTGTGTGTTCCCGTTCATCT	CGCTGGAATGATCTAAGCCCA
NFATc1	CCGTTGCTTCCAGAAAATAACA	TGTGGGATGTGAACTCGGAA
Clcr	CATTCCTGTTACTTGGTTGGC	AGCAATCGACAAGGAGTGAC
DC-STAMP	GACCTTGGGCACCAGTATTT	CAAAGCAACAGACTCCCAAA
ATP6v0d2	GTGAGACCTTGGAA GACCTGAA	GAGAAATGTGCTCAGGGGCT
αv	GCCAGCCCATTGAGTTTGATT	GCTACCAGGACCACCGAGAAG
β3	CCGGGGGACTTAATGAGACCACTT	ACGCCCCAAATCCCACCCATACA
Fas	CCTGCTCCTCATCATTGTGT	TGATCCATGTACTCCTCTCC
FasL	CTGGGTTGTACT TCGTGTATTCC	TGTCCAGTAGTGCAGTAGTTCAA
RelB	CTTTGCCTATGATCCTTCTGC	GAGTCCAGTGATAGGGGCTCT
NFκB1 (p50/p105)	GAAATTCCTGATCCAGACAAAAAC	ATCACTTCAATGGCCTCTGTGTAG
NFκB2 (p52/p100)	CTGGTGGACACATACAGGAAGAC	ATAGGCACTGTCTTCTTTCACCTC
IκBα promoter	AAATCTCCAGATGCTACCCGAGAG	ATAATGTCAGACGCTGGCCTCCAA
TNFα promoter	CCCCAGATTGCCACAGAATC	CCAGTGAGTGAAAGGGACAG
Runx2	GCTTGATGACTCTAAACCTA	AAAAAGGGCCCAGTTCTGAA
Osterix	AGGCACAAAGAAGCCATAC	AATGAGTGAGGGAAGGGT
ATF4	ATGGCGCTCTTCACGAAATC	ACTGGTCGAAGGGGTCATCAA
ALP	AATCGGAACAACCTGACTGACC	TCCTTCCACCAGCAAGAAGAA
HPRT1	CTGGTGAAAAGGACCTCTCGAA	CTGAAGTACTCATTATAGTCAAGGGCAT
SOST	ACCACCCCTTTGAGACCAAAG	GGTCACGTAGCGGGTGAAGT
GAPDH	ACCCAGAAGACTGTGGATGG	CAGTGAGCTTCCCGTTCAG

MTI-Ⅱ macromolecular translocation inhibitor Ⅱ, TRAP tartrate resistant acid phosphatase, MMP matrix metalloproteinase, NFATc1 nuclear factor of activated T cells c1, Clcr calcitonin receptor, DC-STAMP dendritic cell-specifc trans-membrane protein, FasL Fas ligand, NFκB nuclear factor-kappa B, Runx2 runt-related transcription factor 2, ATF4 activating transcription factor 4, ALP alkaline phosphatase, HPRT1 hypoxanthine phosphoribosyltransferase 1, GAPDH glyceraldehyde-3-phosphate dehydrogenase
